# Molecular Characterization of the Complete Genome of Three Basal-BR Isolates of *Turnip mosaic virus* Infecting *Raphanus sativus* in China

**DOI:** 10.3390/ijms17060888

**Published:** 2016-06-04

**Authors:** Fuxiang Zhu, Ying Sun, Yan Wang, Hongyu Pan, Fengting Wang, Xianghui Zhang, Yanhua Zhang, Jinliang Liu

**Affiliations:** College of Plant Sciences, Jilin University, Changchun 130062, China; fengxiangsui2009@163.com (F.Z.); sunying8213@163.com (Y.S.); w1178014711@163.com (Y.W.); panhongyu@jlu.edu.cn (H.P.); wft1001@163.com (F.W.); zhangxianghui@jlu.edu.cn (X.Z.); yh_zhang@jlu.edu.cn (Y.Z.)

**Keywords:** TuMV, complete genome sequence, basal-BR, genetic diversity, *Raphanus sativus*

## Abstract

*Turnip mosaic virus* (TuMV) infects crops of plant species in the family *Brassicaceae* worldwide. TuMV isolates were clustered to five lineages corresponding to basal-B, basal-BR, Asian-BR, world-B and OMs. Here, we determined the complete genome sequences of three TuMV basal-BR isolates infecting radish from Shandong and Jilin Provinces in China. Their genomes were all composed of 9833 nucleotides, excluding the 3′-terminal poly(A) tail. They contained two open reading frames (ORFs), with the large one encoding a polyprotein of 3164 amino acids and the small overlapping ORF encoding a PIPO protein of 61 amino acids, which contained the typically conserved motifs found in members of the genus *Potyvirus*. In pairwise comparison with 30 other TuMV genome sequences, these three isolates shared their highest identities with isolates from Eurasian countries (Germany, Italy, Turkey and China). Recombination analysis showed that the three isolates in this study had no “clear” recombination. The analyses of conserved amino acids changed between groups showed that the codons in the TuMV out group (OGp) and OMs group were the same at three codon sites (852, 1006, 1548), and the other TuMV groups (basal-B, basal-BR, Asian-BR, world-B) were different. This pattern suggests that the codon in the OMs progenitor did not change but that in the other TuMV groups the progenitor sequence did change at divergence. Genetic diversity analyses indicate that the *PIPO* gene was under the highest selection pressure and the selection pressure on *P3N-PIPO* and *P3* was almost the same. It suggests that most of the selection pressure on *P3* was probably imposed through *P3N-PIPO*.

## 1. Introduction

*Turnip mosaic virus* (TuMV) is a member of the genus *Potyvirus* and possesses an exceptionally broad host range in terms of plant genera and families compared to any other potyvirus; it is known to infect at least 318 species of 156 genera belonging to 43 families of plants representing many arable, vegetable and ornamental crops, especially in the family *Brassicaceae* [1,2]. TuMV was ranked second only to *Cucumber mosaic virus* as the most important virus infecting field-grown vegetables in a survey of virus diseases in 28 countries and regions [3].

TuMV forms flexuous filamentous particles 700–750 nm in length, each of which contains a single copy of the genome, which is a single-stranded, positive-sense RNA molecule of about 10,000 nt. The virion RNA is infectious and serves as both the genome and viral messenger RNA. The genomic RNA is translated into one large polyprotein which is subsequently processed by the action of three viral-encoded proteinases (Pl, HC-Pro and NIa-Pro) into functional products [4]. Furthermore, P3N-PIPO is expressed by a +2 ribosomal frameshift within the P3 ORF and probably acts as a movement protein [5,6].

TuMV is a highly variable virus. The world population of TuMV has probably been more thoroughly sampled and sequenced than that of any other potyviruses [7]. Studies of the genetic and molecular variability of viruses help to provide understanding of some important features of their biology, like changes in virulence, host adaptation, geographical ranges, their new emergence and so on [2,7,8,9,10,11,12,13]. The present worldwide population of TuMV is evolving and diverging rapidly. TuMV is spread by polyphagous aphids as well as in seed and infected plant materials. On average, the nucleotide evolutionary rate of TuMV is around 10^−3^ substitutions per site per year [7]. In previous studies of the molecular evolution of TuMV, the virus was found to have four phylogenetic lineages (basal-B, basal-BR, Asian-BR, world-B) [12]. Recently, Nguyen *et al.* reported that TuMV diverged from a closely related TuMV-like virus (TuMV-OMs) from wild orchids in Europe about 1000 years ago. It was viewed as a significant host switch between monocotyledonous and dicotyledonous plant lineages [7]. It was supposed that non-unique combinations of transient viral genomic single nucleotide polymorphisms (SNPs) allowed the host switch to occur [9]. The five host-infecting types are mostly congruent with the phylogenetic groupings [7,11,14]. Isolates from host type OM infect some *Brassicaceae* plants but not *Brassica* spp. plants. Isolates from host type [(B)] infect *Brassica* plants latently and occasionally but not *Raphanus* plants. Isolates from host type [B] infect most *Brassica* species systemically (systemic mosaic symptoms) but do not infect *Raphanus* plants. Isolates from host type [B(R)] infect most *Brassica* systemically (systemic mosaic symptoms) and occasionally infect *Raphanus* plants latently. Isolates from host type [BR] infect both *Brassica* and *Raphanus* plants systemically (systemic mosaic symptoms). The paraphyletic basal-B cluster of [(B)] pathotype isolates is the most variable. The world-B cluster is the more variable and widespread cluster. The basal-BR and the Asian-BR clusters of the [BR] pathotype isolates are less variable [12,15]. Most European isolates do not infect *Raphanus*, whereas Asian isolates infect both *Brassica* and *Raphanus*.

Potyviruses have spread throughout much of the subtropical and temperate zones of the world. Phylogeographic analysis of the potyviruses indicates that the genus originated in western Eurasia and/or North Africa, and their recent adaptive radiation has involved many emergences [9,16]. Like other potyviruses, TuMV originated in Europe and spread to other parts of the world, and that the basal-BR population, in a state of sudden expansion, has recently evolved in East Asia [8,12,13,17]. Studies of the genetic structure of plant virus populations have shown that the evolution of virus populations is shaped by founder effects, selection and recombination [14]. As in other potyviruses, recombination is a frequent event in the evolution of TuMV. TuMV isolates from China and Japan are part of the same population but are a discrete lineage. TuMV populations in Vietnam are clustered in the world-B group with clear local founder effects compared to that of Chinese and Japanese isolates and there have been no basal-BR isolates [11]. The basal-BR isolates were first reported in 2000 in Japan, when they were at an epidemic situation nationwide [2].

Previous studies showed that TuMV isolates in China belonged to the world-B and Asian-BR groups [2,12,13,18,19]. However, we first detected the existence of basal-BR isolates in China in the years 2004–2005 [17]. We then reported the full genomic sequences of the first two basal-BR isolates and analyzed their recombination pattern and genetic diversity [8]. In the study reported here, the complete genome sequences of three TuMV isolates collected from radish from Shandong and Jilin Provinces in China were determined. We analyzed their phylogenetic relationships, recombination pattern and genetic diversity, together with 30 other previously reported representative TuMV isolates collected from different hosts around the world. The results showed that the three isolates were clustered to the basal-BR group and had no “clear” recombination. Genetic diversity analyses indicate that the selection pressure on *PIPO* was the highest and that of *P3N-PIPO* and *p3* was almost the same.

## 2. Results

### 2.1. Genome Structure and Characterization of Putative Polyproteins

The complete genome sequences of the CCLB, LWLB and WFLB14 isolates were all 9833 nt (excluding the 3′ poly(A) tail) and were allocated the accession numbers of KR153038, KR153039 and KR153040. The base composition of the three isolate’s genomic RNA were not significantly different, with the following nucleotide percentages: adenine 31.75%–31.76%, cytosine 21.56%–21.64%, guanine 24.14%–24.16% and uracil 22.46%–21.54%. The genome contained one large ORF that coded for a polyprotein of 3164 residues and was flanked by 5′ and 3′ untranslated regions (UTR) of 129 and 209 nt, respectively. The 5′-UTR contained the conserved sequences ACAACAU (positions 20–26) and UCAAGCA (positions 67–73), corresponding to potyboxes “a” and “b”, respectively, which probably plays an important role in transcription initiation [20,21]. The first inframe translation start codon AUG (130–132) of the three isolates genomic RNA was situated in the appropriate context of AGCAAUGGC (CCLB and LWLB isolates) and AGAAAUGGC (WFLB14 isolate), which were similar to the corresponding sequence AACAAUGGC in plants [22]. The −3 and +4 nucleotides were both purines that might be required for initiation of translation [23].

**Figure 1:**
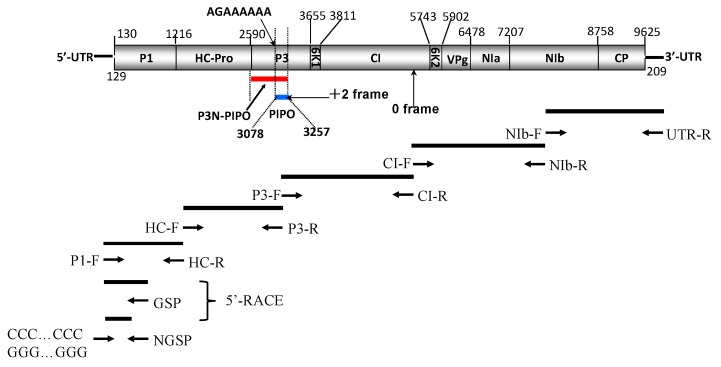
Schematic presentation of TuMV (*Turnip mosaic virus*) full genome cloning strategies. 5′-UTR = 5′-untranslated region; P1 = protein 1; HC-Pro = helper component proteinase; P3 = protein 3; PIPO = Pretty Interesting Potyviridae ORF; 6K1 = peptide 1; CI =cylindrical inclusion protein; 6K2 = peptide 2; VPg = viral genome-linked protein; NIa-Pro= nuclear inclusion a (proteinase); NIb = nuclear inclusion b (viral replicase); CP = coat protein; 3′-UTR = 3′-untranslated region.

As predicted by Adams *et al.* [24], nine putative protease cleavage sites were identified in the CCLB, LWLB and WFLB14 polyprotein at amino acid positions 362, 820, 1175, 1227, 1871, 1924, 2116, 2359 and 2876 that give rise to ten mature proteins: P1, HC-Pro, P3, 6K1, CI, 6K2, VPg, NIa-Pro, NIb and CP. The cleavage sites for P1 and HC-Pro were Y/S and G/G, respectively, and those for NIa-Pro were Q/A, Q/T, Q/N, E/A, E/S, Q/T and Q/A (Figure 1, Table 1).

Comparing amino acid sequences of CCLB, LWLB and WFLB14 with other potyviruses, conserved motifs in each of these proteins were identified. In HC-Pro, the ^414^KITC^417^ and ^672^PTK^674^ were important motifs involved in aphid transmission [25]; the motif ^543^FRNK^546^ has been shown to be involved in symptom formation [26] and suppression of RNA silencing [27]; the motifs ^654^CCCVT^658^, ^679^IGN^681^ and ^706^C-X_72_-H^779^ have been implicated in virus cell-to-cell movement [28], genome amplification [29] and proteinase activity [30], respectively. The NTP-binding motif ^1313^GAVGSGKS^1320^ in the CI protein was involved in helicase activity [31]. Additionally, the ^1480^LVYV^1483^, ^1531^VATNIIENGVTL^1542^ and ^1575^GERIQRLGRVGR^1586^ motifs might also be characteristic of helicase proteins [32]. The conserved tyrosine residue (Y) in the context ^1986^MYGF^1989^ has been shown to be essential for linking VPg to genomic RNA [33]. In NIa-Pro, ^2162^H-(X)_34_-D-(X)_69_-C-(X)_15_-H^2283^ was a conserved motif associated with proteinase activity [34]. In NIb, the conserved motif ^2611^FDSS^2614^ was located 266 aa upstream of the putative NIb/CP cleavage site [35], and ^2710^GDD^2712^ was necessary for RNA polymerase activity and NTP binding [35]. Like the CPs of most potyviruses, these three TuMV isolates also carried the ^2882^DAG^2884^ motif interacting with the PTK of HC-Pro as an important factor related to aphid transmission, and the ^3054^R-(X)_43_-D^3098^ motif associated with virus movement [36]. In addition, The three consensus motifs, ^3013^MVWCIENGTSP^3023^, ^3096^AFDF^3099^ and ^3116^QMKAAA^3121^, were also found in the CPs of the CCLB, LWLB and WFLB14 isolates [35].

The recently described ORF coding the putative protein PIPO [6] was identified within the P3 ORF expressed by a +2 ribosomal frameshift, starting from a G_1_A_4_ motif at position 3076 (Figure 1). This motif was distinct from the highly conserved motif G_1–2_A_6–7_ that is known for other potyviruses [6] and ended with a UAA termination codon at position 3258–3261.

### 2.2. Percentage Identity

Pairwise comparisons of the CCLB, LWLB and WFLB14 genome sequences with those of 30 other TuMV isolates available in sequence databases show that the most closely related isolate is DEU4 (AB701701), which was the BR pathotype from Germany, sharing 95.0%, 94.7% and 94.8% nucleotide identity, respectively (Table 2). The nucleotide and amino acid identities of each gene in these three isolates compared with those of others is shown in Table 2, and the isolates shared the highest identities with genes from Cal1 (AB093601), DEU4 (AB701701), PV0104 (AB093603), ITA8 (AB701725) and USA6 (AB701741).

### 2.3. Phylogenetic Relationships

**Figure 2:**
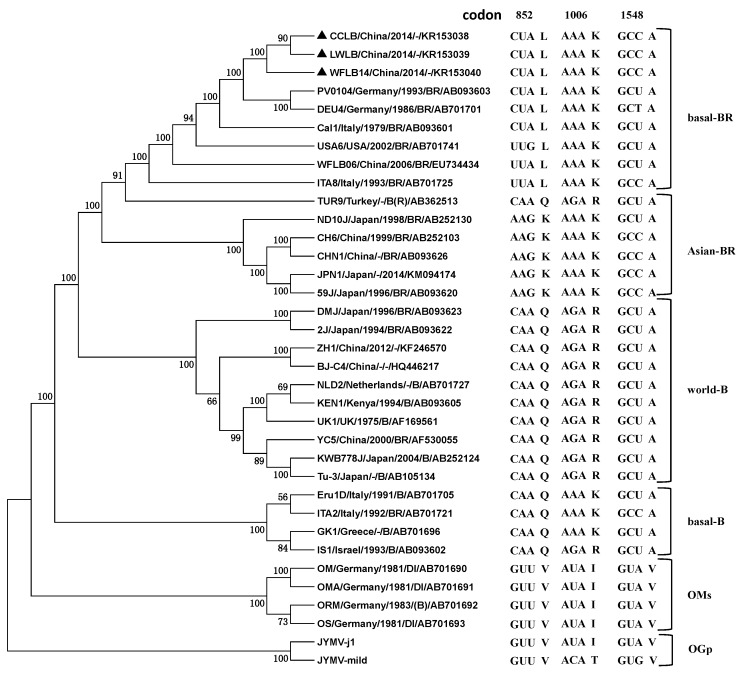
A maximum likelihood tree showing the relationships inferred from the complete genome sequences of 33 viruses of the TuMV group, together with those of isolates of JYMV as an outgroup (OGp). The branches are horizontal and show, for each genome, the codons at sites 852, 1006 and 1548, numbered as in the alignment used. Bootstrap values (%) more than 60 are listed; nodes with <50% bootstrap support have been collapsed. Isolates are indicated in the tree by isolate name/geographical origin/origin of host/pathotype. “-“ indicates the corresponding information is not available.

To estimate the phylogenetic relationships among the TuMV isolates and the outgroups, the complete genome sequences of 33 TuMV isolates, including the three isolates determined in this work, were subjected to phylogenetic analyses, with two *Japanese yam mosaic virus* (JYMV) isolates (AB016500 and AB027007) sharing the highest identities with TuMV as the outgroup (Table 3, Figure 2). The maximum likelihood (ML) tree showed that these TuMV isolates were clustered to five lineages corresponding to basal-B, basal-BR, Asian-BR, world-B and OMs, which was consistent with previous reports [7,9]. The CCLB, LWLB and WFLB14 were clustered to the basal-BR group. The phylogenetic trees estimated for the individual *P1* and *CP* genes of the 33 isolates were very similar with the results above (data not shown).

In a recent report, it was showed that the “emergence” of TuMV was probably a “gene-for-quasi-gene” event based on *in vivo* and *in silico* studies [9]. According to this, conserved amino acids changed between the group were also found in 35 sequences, including two JYMV isolates (outgroup) using the clustal W program (Figure 2). At codon positions 852 and 1006, their OGp and OMs amino acids were the same (^852^V, ^1006^I), and the other TuMV groups (basal-B, basal-BR, Asian-BR, world-B) were different (^852^K /^852^Q/^852^L, ^1006^K/^1006^R). This asymmetric phylogenetic pattern was called the XXY pattern, which suggests that, at the divergence, the amino acids in the OM progenitor did not change, but in the TuMV other groups the progenitor did. In both sites, the encoded amino acids changed from hydrophobic to polar amino acids. Codon 852 encodes an amino acid at the N-terminal end of the P3 protein and Codon 1006 is translated to an amino acid in the C-terminal third of the P3 protein and near the 5′ terminal third of the PIPO ORF. Amino acid 1548 in the middle of the CI protein sequence was unique to all isolates of OGp and OMs (^1548^V), while the alternate (^1548^A) was conserved in the other TuMV groups. The results in this work were consistent with that of Gibbs *et al.* [9]. At three codon sites (852, 1006, 1548), CCLB, LWLB and WFLB14 were all ^852^L^1006^K^1548^A, which were conserved in isolates of the basal-BR group (Figure 2).

### 2.4. Recombination Analysis

The polyprotein-encoding gene sequences of 33 isolates from the public DNA sequence databases were screened for possible recombination events in isolates CCLB, LWLB and WFLB14 and assessed for evidence of recombination using an RDP version 4 software package, PHYLPRO version 1 and SISCAN version 2. Only three out of 33 genomes (9.1%) showed evidence of recombination. The CCLB, LWLB and WFLB14 isolates in this study had no “clear” recombination. Three recombinant isolates (CHN1, ND10J and 59J) were all intralineage recombinants of Asian-BR and world-B isolates, most with CH6 of Asian-BR as the major parent and 2J or KWB778J of world-B as the minor parent. Most recombination sites were located in *P1*, *CI*, *6K2* and *CP*, which were hotspots of recombination in TuMV [8,13,37] (Table 4).

### 2.5. Genetic Distance and Selection Pressure

Genetic distances of the 33 isolates within and between groups were calculated by the K2P methods in MEGA version 6 [38] (Table 5). It showed that the genetic distance within the basal-B group was the largest (0.185 ± 0.004), two times that of the basal-BR group, in which CCLB, LWLB and WFLB14 were clustered. Genetic distances within the group were significantly smaller than those between groups, indicating that the groupings are correct.

Each gene was checked to determine the direction of mutation of 16 TuMV isolates in Chinese and Japanese populations in their *d*_N_/*d*_S_ substitution rates using the codeml program and PBL method (Table 6). It was found that the values of the *d*_N_/*d*_S_ ratio were always <1 and differed considerably in different genomic regions, indicating that there was selection against most amino acid changes, namely, “negative selection” or “purifying selection”, in most of these regions. The largest *d*_N_/*d*_S_ ratio was for the *PIPO* gene. However, the *d*_N_/*d*_S_ ratio of *P3N-PIPO* and *P3* were almost the same, each only one quarter that of the *PIPO* gene. This indicates that most of the selection pressure in *P3* was imposed by *P3N-PIPO*. In addition, the *d*_N_/*d*_S_ ratios for the *P1* gene was also larger than that of all other genes except *PIPO*. This result was helpful in understanding that *P1* and *P3* are the most variable genes in the TuMV genome [10]. The *d*_N_/*d*_S_ ratios for the Chinese and Japanese populations and for the different phylogenetic lineages were not significantly different for any of the genes analyzed by the two methods.

## 3. Discussion

Here, we reported the complete genome sequences of three Chinese TuMV isolates infecting *Raphanus sativus* that were grouped to basal-BR lineage according to their molecular characteristics. Basal-BR is a recent “emerged” branch of the population in East Asia, which was in a state of sudden expansion [8,11,12,13,17]. In China, since the first report of the basal-BR lineage isolates [17], the population of basal-BR isolates increased rapidly and showed characteristics of the founder effect [8]. In this study, CCLB, LWLB and WFLB14 genome sequences shared their highest identities with isolates from Eurasian countries (Germany, Italy, Turkey and China) and were clustered in the basal-BR group (Table 2, Figure 2) which was consistent with the (delete possible) interpretations that TuMV originated in western Eurasia and spread to other parts of the world [12].

Recently, a sister lineage of TuMV-like potyviruses (TuMV-OM) was identified from European orchids (Orchis militaris, Orchis morio and Orchis simia), from which TuMV diverged about 1000 years ago [7]. A virus emergence involving a major host switch would probably result in significant genomic changes, especially in the emergent lineage [9]. In this work, conserved amino acids changed between groups were found in 35 sequences, including two JYMV isolates (OGp) (Figure 2). The OGp and OMs groups codons were the same at three codon sites (852, 1006, 1548), and their TuMV other groups (basal-B, basal-BR, Asian-BR, world-B) were different. This pattern suggests that the codon in the OMs progenitor did not change but that in the TuMV other groups the progenitor did change at the divergence. Gibbs described this “emergence” of TuMV as probably a “gene-for-quasi-gene” event [9]. Codon 852 and 1006 are translated to amino acids in the P3 protein, and the amino acid 1548 is in the middle of the CI protein sequence. In previous studies, the *P3* and *CI* genes of TuMV, together with the small *6K2* and *VPg* genes, were identified to be involved in host determination [9,39,40,41].

The degree of selection pressure in genes can be estimated by calculating the *d*_N_/*d*_S_ ratios, which provide evidence of strong selection against amino acid change as a driving force for TuMV evolution [13,15,42,43]. In previous results, it was shown that Chinese TuMV isolates were under negative or purifying selection according to the whole ORF and other genes [8,9,17]. In this work, we also checked gene-by-gene to see whether there were significant differences between the Chinese and Japanese populations in their *d*_N_/*d*_S_ substitutions (Table 6). Surprisingly, the selection pressure on *PIPO* was the highest, not *P1*, which was reported to be the highest in previous studies [8,13,15]. This may be interpreted that *PIPO* was recently described as a new ORF encoded within the genome of the *Potyviridae* family [6], and the selection pressure on *PIPO* was not examined in that search. Additionally, we also found that the selection pressure on *P3N-PIPO* and *P3* was almost the same, but only one quarter that of *PIPO*. The *PIPO* ORF is embedded within the *P3* cistron and is translated in the +2 reading frame relative to the potyviral long ORF as the P3N-PIPO fusion protein. So it suggests that most of the selection pressure on *P3* was imposed by *P3N-PIPO* and seems not to account for the presence of alternative stop codons in PIPO ORF [44]. This is hypothesized to be associated with the function of P3N-PIPO in cell-to-cell movement and overcoming host resistance [5,42,45,46]. In addition, the selection pressure on the *P1* gene was (delete also) larger than that of all other genes except *PIPO*. The higher selection pressure on *P1*, *P3* and *P3N-PIPO* might provide an evolutionary force to host dependence and adaptation [5,9,13,15].

TuMV isolates of basal-BR are prevalent and expanding rapidly in China since the first report of their existence in 2005. More full genome sequences of TuMV isolates of basal-BR in China were identified, which made it possible to further understand the genetic diversity of TuMV comprehensively. Our results provided useful information about the evolution and genetic conservation of TuMV. It will also be important in the future to study the pathogenic mechanism of TuMV and the resistance of cruciferous crops to TuMV isolates.

## 4. Materials and Methods

### 4.1. Virus Samples, RNA Extraction and Sequencing

Three samples with typical symptoms of viral diseases were collected from radish from Shandong and Jilin Provinces, which we named WFLB14, LWLB and CCLB. All the isolates were sap-inoculated to *Chenopodium amaranticolor* and serially cloned through single lesions at least three times. They were propagated in *Brassica rapa* plants.

The viral RNAs were extracted from purified virions with an Invitrogen Trizol Kit following the instructions of the manufacturer. The RNAs were reverse-transcribed with UTR-R, a primer that was complementary to poly (A) (Figure 1, Table 7). Most parts of the genomes were amplified by PCR (Platinum^®^ Taq DNA Polymerase, Invitrogen, Carlsbad, CA, USA) with forward primers and reverse primers, which will be provided upon request, and designed according to the conserved region and newly determined genomic sequences (Figure 1, Table 7). The 5′-proximal part was obtained using GSP and NGSP primers (Figure 1, Table 7) with the 5′-Rapid Amplification of cDNA Ends (5’-RACE) method, as described in an earlier study [47].

The amplification products were ligated to the vector pMD18-T (TaKaRa Biotechnology Dalian Co., Ltd., Dalian, China), which was confirmed by PCR and restriction enzyme digestion before sequencing by an ABI PRISMTM 377 DNA Sequencer. Nucleotide sequences from each isolate were determined using at least four overlapping independent RT-PCR products for each region to cover the complete genome. At least six clones for the fragments obtained from the 5′-RACE were sequenced. The determined sequences were assembled with DNAMAN (Lynnon Biosoft, Vandreuil, QC, Canada) and DNASTAR Lasergene (DNASTAR, Inc., Madison, WI, USA).

### 4.2. Phylogenetic Analyses

To estimate the phylogenetic relationships among the TuMV isolates and the outgroups, we aligned the 33 complete genome sequences (Table 3) using the clustal W program [48] and constructed the Phylogenetic tree using the maximum likelihood method in the MEGA version 6 [38]. Statistical significance of tree branching was tested by performing 1000 bootstrap replications. Two *Japanese yam mosaic virus* (JYMV) isolates (JYMV-j1 and JYMV-mild) [49,50] with known complete genomic sequences were used as the outgroup (OGp), because BLAST searches had shown them to be most closely and consistently related to those of TuMV. In order to identify sequence changes between groups, codons of interest were examined in an alignment of 33 sequences using the clustal W program [9,48].

### 4.3. Recombination Analyses

According to the Phylogenetic trees constructed using different genes of TuMV, we initially confirmed the probable instances of recombination by analyses of different isolates clustered into the lineages. All sequences were aligned using the clustal X program [48] and determined using a combination of methods in the RDP version 4 software package [51], namely RDP [51], GENECONV [52], BOOTSCAN [53], CHIMEARA [54] and MAXCHI [55]. All isolates that had been identified as likely recombinants by the programs in RDP version 4, supported by three different methods with an associated *p-*value of <1.0 × 10^−6^, were re-checked using the original PHYLPRO version 1 [56] and SISCAN version 2 [57].

### 4.4. Genetic Distance and Selection Pressure

Genetic distances of all the isolates within and between groups were calculated by Kimura2-parameter (K2P)methods in MEGA version 6 [38]. Non-synonymous (*d*_N_) and synonymous (*d*_S_) differences that correlated with phylogenetic relationships were estimated using the codeml program of PAML version 4 [58] and the Pamilo-Bianchi-Li (PBL) method assembled in MEGA version 6 [38]. The *d*_N_/*d*_S_ ratios, representing selection pressure in evolution for each protein-encoding region of TuMV sub-populations of different collection regions, were calculated using the Pamilo-Bianchi-Li method in MEGA version 6 [38].

## Figures and Tables

**Table 1:** Genome structure and nine putative protease cleavage sites in polyproteins of CCLB, LWLB and WFLB14.

Region (Gene)	Start–End Site	Size in nt/aa	Cleavage Site (C-Terminus)
5′-UTR	1–129	129/–	
*P1*	130–1215	1086/362	Y/S
*HC-Pro*	1216–2589	1374/458	G/G
*P3*	2590–3654	1065/355	Q/A
*6K1*	3655–3810	156/52	Q/T
*CI*	3811–5742	1932/644	Q/N
*6K2*	5743–5901	159/53	E/A
*VPg*	5902–6477	576/192	E/S
*NIa-Pro*	6478–7206	729/243	Q/T
*NIb*	7207–8757	1551/517	Q/A
*CP*	8758–9621	864/288	-
3′-UTR	9625–9833	209/-	-

**Table 2:** Highest identities of individual CCLB, LWLB and WFLB14 genes with other TuMV isolates at nucleotide (nt) and amino acid (aa) levels.

Region (Gene)	CCLB			LWLB			WFLB14		
	Isolate Name	nt (%)	aa (%)	Isolate Name	nt (%)	aa (%)	Isolate Name	nt (%)	aa (%)
*P1*	Cal1	95.3	94.2	Cal1	94.7	93.4	Cal1	95.5	94.5
*HC-Pro*	DEU4	97.0	99.8	DEU4	96.9	99.8	DEU4	96.7	99.8
*PIPO*	PV0104/DEU4	99.5	98.4	PV0104/DEU4	99.5	98.4	PV0104/DEU4	99.5	98.4
*P3*	PV0104/DEU4	97.1	98.6	PV0104/DEU4	96.9	98.6	PV0104/DEU4	96.8	98.6
*6K1*	PV0104/DEU4	99.4	100.0	PV0104/DEU4	98.1	100.0	PV0104/DEU4	99.4	100.0
*CI*	DEU4	98.7	99.8	DEU4	98.6	99.8	DEU4	98.3	99.8
*6K2*	PV0104/DEU4	98.1	100.0	PV0104/DEU4	96.9	96.2	Cal1	98.1	100
*NIa-VPg*	Cal1	95.5	99.0	Cal1	95.7	99.0	Cal1	95.8	99.5
*NIa-Pro*	ITA8	91.5	98.8	ITA8	90.5	98.8	ITA8	90.9	98.8
*NIb*	USA6	93.6	98.8	USA6	93.3	98.6	USA6	93.5	98.8
*CP*	PV0104/DEU4	96.6	99.0	PV0104/DEU4	96.5	99.0	PV0104/DEU4	96.6	98.6
Polyprotein	DEU4	94.9	98.1	DEU4	94.6	97.9	DEU4	94.7	98.0
Genome Sequence	DEU4	95.0	-	DEU4	94.7	-	DEU4	94.8	-

**Table 3:** TuMV isolates analyzed in this study ^a^.

Accession Number	Isolates	Original Host	Location	Pathotype ^b^	Year of Collection
AB093622	2J	*Brassica pekinensis*	Japan	BR	1994
AB093620	59J	*Raphanus sativus*	Japan	BR	1996
AB252097	AD855J	*Raphanus sativus*	Japan	BR	2002
AB252099	AKD161J	*Raphanus sativus*	Japan	BR	1998
AB252102	AT181J	*Eustoma russellianum*	Japan	BR	<1998
HQ446217	BJ-C4	cruciferous plants	China	Unknown	1985–1987
AB093601	Cal1	*Calendula officinalis*	Italy	BR	1979
AB252103	CH6	*Raphanus sativus*	China	BR	1999
AB252104	CHK16	*Raphanus sativus*	China	BR	2000
AB252105	CHL13	*Raphanus sativus*	China	BR	1999
AB093626	CHN1	*Brassica* sp.	China	BR	<1980
AB093614	CP845J	*Calendula officinalis*	Japan	BR	1997
AB701701	DEU4	*Lactuca sativa*	Germany	BR	1986
AB093623	DMJ	*Raphanus sativus*	Japan	BR	1996
AB701705	Eru1D	*Eruca sativa*	Italy	B	1991
AB252109	FKD001J	*Raphanus sativus*	Japan	BR	2000
AB701696	GK1	*Matthiola incana*	Greece	B	<1989
AB252118	H1J	*Raphanus sativus*	Japan	BR	1996
AB093627	HRD	*Raphanus sativus*	China	BR	1998
AB093602	IS1	*Allium ampeloprasum*	Israel	B	1993
AB701721	ITA2	*Cheiranthus cheiri*	Italy	BR	1992
AB701725	ITA8	*Abutilon* sp.	Italy	BR	1993
KM094174	JPN 1	*Raphanus sativus*	Japan	Unknown	2014
AB093605	KEN1	*Brassica oleracea*	Kenya	B	1994
AB252124	KWB778J	*Brassica oleracea*	Japan	B	2004
AB252125	KWB779J	*Brassica rapa*	Japan	BR	2004
AB252130	ND10J	*Raphanus sativus*	Japan	BR	1998
AB701727	NLD2	*Brassica oleracea*	Netherlands	B	<1995
AB701690	OM	*Orchis militaris*	Germany	DI ^c^	1981
AB701691	OMA	*Orchis militaris*	Germany	DI	1981
AB701692	ORM	*Orchis morio*	Germany	(B)	1983
AB701693	OS	*Orchis simia*	Germany	DI	1981
AB093603	PV0104	*Lactuca sativa*	Germany	BR	1993
AY134473	RC4	*Zantedeschia* sp.	China	BR	2000
AB093615	TD88J	*Raphanus sativus*	Japan	BR	1998
AB105134	Tu-3	*Brassica oleracea*	Japan	B	Unknown
AB362513	TUR9	*Raphanus sativus*	Turkey	B(R)	<2007
AF169561	UK1	*Brassica napus*	UK	B	1975
AB701741	USA6	*Raphanus sativus*	USA	BR	2002
EU734434	WFLB06	*Raphanus sativus*	China	BR	2006
AF530055	YC5	*Zantedeschia* sp.	China	BR	2000
KF246570	ZH1	*Phalaenopsis* sp.	China	Unknown	2012
KR153038	CCLB	*Raphanus sativus*	China	Unknown	2014
KR153039	LWLB	*Raphanus sativus*	China	Unknown	2014
KR153040	WFLB14	*Raphanus sativus*	China	Unknown	2014

^a^ All the data were from the National Center for Biotechnology Information (NCBI, http://www.ncbi.nlm.nih.gov/); ^b^ Pathotype [B] isolates, infect most *Brassica* species systemically but do not infect *Raphanus* plants. Pathotype [(B)] isolates, infect *Brassica* plants latently and occasionally but not *Raphanus* plants. Pathotype [BR] isolates , infect both *Brassica* and *Raphanus* plants systemically. Pathotype [B(R)] isolates, infect most *Brassica* systemically and occasionally infect *Raphanus* plants latently; ^c^ DI, Difficult to infect brassica plants.

**Table 4:** Recombination sites and possible parent-like isolates.

Isolate	Recombination Region	“Parential-Like” Isolate		Type of “Recombinant”	Recombination Detection	
		Major	Minor		Methods *	*p*-Value
CHN1	nt 8872-9776 (5′-UTR-*P1*)	CH6	2J	Asian-BR × world-B	RGCS	1.334 × 10^−8^
ND10J	nt 143-723 (5′-UTR-*P1*)	CH6	KWB778J	Asian-BR × world-B	RGMCS3	1.410 × 10^−14^
	nt 4598-5983 (*VPg-CP*)	59J	DMJ	Asian-BR × world-B	RGBMCS3	1.608 × 10^−25^
	nt 9133-9759 (*VPg-CP*)	CH6	2J	Asian-BR × world-B	RGCS	1.334 × 10^−8^
59J	nt 142-742 (*HC-Pro-P3*)	CH6	KWB778J	Asian-BR × world-B	RGMCS3	1.410 × 10^−14^
	nt 9174-9759 (*HC-Pro-P3*)	CH6	2J	Asian-BR × world-B	RGCS	1.334 × 10^−8^

* The programs supporting recombination events. RGBMCS3 represent RDP, GENECONV, BootScan, MaxChi, Chimaera, SiScan and 3Seq, respectively. The program that had the greatest *p*-value is underlined.

**Table 5:** The genetic distance of within and between populations and sub-populations.

Group	OMs	world-B	Asian-BR	basal-BR	basal-B
OMs	0.003 ± 0.000				
world-B	0.278 ± 0.005	0.042 ± 0.001			
Asian-BR	0.286 ± 0.005	0.175 ± 0.003	0.037 ± 0.001		
basal-BR	0.281 ± 0.004	0.182 ± 0.003	0.158 ± 0.004	0.092 ± 0.002	
basal-B	0.283 ± 0.005	0.219 ± 0.004	0.222 ± 0.004	0.217 ± 0.003	0.185 ± 0.004

**Table 6:** Nucleotide diversity of each coding gene of TuMV isolates collected from China and Japan.

Gene Name	China			Japan		
	*d*_N_	*d*_S_	*d*_N_/*d*_S_	*d*_N_	*d*_S_	*d*_N_/*d*_S_
*P1*	0.097 (±0.009)	0.519 (±0.039)	0.187	0.060 (±0.007)	0.322 (±0.029)	0.186
*HC-Pro*	0.016 (±0.003)	0.854 (±0.083)	0.019	0.015 (±0.003)	0.716 (±0.094)	0.021
*P3*	0.065 (±0.008)	0.705 (±0.057)	0.092	0.064 (±0.007)	0.519 (±0.055)	0.123
*P3N-PIPO*	0.055 (±0.009)	0.594 (±0.067)	0.093	0.051 (±0.009)	0.481 (±0.066)	0.106
*PIPO*	0.047 (±0.013)	0.105 (±0.038)	0.448	0.050 (±0.016)	0.133 (±0.045)	0.375
*6K1*	0.015 (±0.008)	0.769 (±0.151)	0.020	0.017 (±0.009)	0.801 (±0.211)	0.021
*CI*	0.012 (±0.002)	0.567 (±0.032)	0.021	0.011 (±0.002)	0.413 (±0.034)	0.027
*6K2*	0.048 (±0.015)	0.546 (±0.104)	0.088	0.030 (±0.011)	0.280 (±0.086)	0.107
*VPg*	0.042 (±0.008)	0.614 (±0.069)	0.068	0.039 (±0.008)	0.631 (±0.085)	0.062
*NIa-Pro*	0.013 (±0.003)	0.618 (±0.058)	0.021	0.008 (±0.003)	0.471 (±0.064)	0.017
*NIb*	0.013 (±0.003)	0.451 (±0.032)	0.029	0.013 (±0.002)	0.400 (±0.036)	0.033
*CP*	0.017 (±0.004)	0.240 (±0.027)	0.071	0.012 (±0.003)	0.175 (±0.024)	0.069

Numbers in parenthesis represent standard deviation.

**Table 7:** Primers used for amplifying the complete genomic sequences of TuMV.

Primer	Sequence (5′→3′)
P1-F	AAAAATATAAAAACTCAACACAACATACACAAAACGA
HC-R	CTGTCGAAGCCTTTCCARAAGT
HC-F	ACTTYTGGAAAGGCTTCGACAG
P3-R	CGCTGTATCTGCCGCCTAAATCG
P3-F	CGATTTAGGCGGCAGATACAGCG
CI-R	TCCYTCAAGCACTGATATGTTCTC
CI-F	GAGAACATATCAGTGCTTGARGGA
NIb-R	TCTTCYTTCATCTCRGGTGTGAACTC
NIb-F	GAGTTCACACCYGAGATGAARGAAGA
UTR-R	TTTTTTTTTTTTTTTTTTGTCCCTTGCATCCTATCAAATG
5′-RACE-QT	CCAGTGAGCAGAGTGACGAGGACTCGAGCTCAAGCTTTTTTTTTTTTTTTTT
5′-RACE-QO	CCAGTGAGCAGAGTGACG
5′-RACE-QI	GAGGACTCGAGCTCAAGC
GSP	AGCTGCGGCTTCCCTGAGGCTA
NGSP	TCCCAAATTGTACCATTCCGGTG

R = A, G; Y = C, T.
